# Supplementary material for: Machine Learning Analysis of Naïve B-Cell Receptor Repertoires Stratifies Celiac Disease Patients and Controls
Source: Front Immunol. 2021 Mar 10;12:627813. doi: 10.3389/fimmu.2021.627813 (PMC8006302; doi:10.3389/fimmu.2021.627813)
Supplement: Supplementary file 1 [file Data_Sheet_1.PDF]

Supplementary Material

1 SUPPLEMENTARY TABLES

Feature name	Feature explanation	Example
•V,D (family usage)	Frequency of specific V/D family gene in subject repertoire.	V5, D2
•V,D,J (gene usage)	Frequency of specific V/D/J gene.	V5-51, D2-1 ,J6
•V,D,J (allele usage)	Frequency of specific V/D/J allele gene.	V5-51*02, J6*01
•V-J	Frequency of combinatorial joining between V and J family in the subject repertoire.	V2_J6
•JL (Junction Length)	The length of the junction region in the antibody. A frequency column for each functional length. (JL %%3 =0)	81-JL
•V-J-JL	Frequency of combinatorial joining between V family, J family and junction length.	V5_J6_30
•DseqL	Represent the nucleotides sequence length of the Divergence gene (D gene/D family).	D2_9_DseqL, D1_12_DseqL
•FUNC_F	Frequency of non-functional antibodies in the repertoire.<br># Functionality was determined based on parameters conditions: (INDEL=F,IN_FRAME=T, STOP=F, FUN=T, JUNCTION_LENGTH %%3 =0)	
•IgD	Frequency of IgD isotypes in subject repertoire.<br># IgD and IgM are different types of isotypes in a naïve repertoire.	

Table S1. This table lists examples of the extracted sequence annotation based features

	Clusters	Sequence annotation	Atchley Descriptors of AA-Kmers
**LR**	0.76	0.39	-
**MIL**	-	-	0.59
**SVM**	0.68	0.48	-
**KNN**	0.71	0.37	-
**Representation description**	Grouped antibody sequences by identical V and J gene annotations and CDR3 sequence similarity	Repertoire annotations descriptors, including usage of V, D, and J genes, alleles, junction lengths, frequency of combinatorial joining of genes, and isotype usage.	Biophysicochemical descriptors of overlapping CDR3 subsequences of equal length.

Table S2. Average F1-score comparison between different models depending on the representation of naïve antibody repertoires
